# Supplementary material for: Tofu Whey Wastewater as a Beneficial Supplement to Poultry Farming: Improving Production Performance and Protecting against Salmonella Infection
Source: Foods. 2022 Dec 23;12(1):79. doi: 10.3390/foods12010079 (PMC9818456; doi:10.3390/foods12010079)
Supplement: Supplementary file 1 [file foods-12-00079-s001.zip › foods-2084202-SI.pdf]

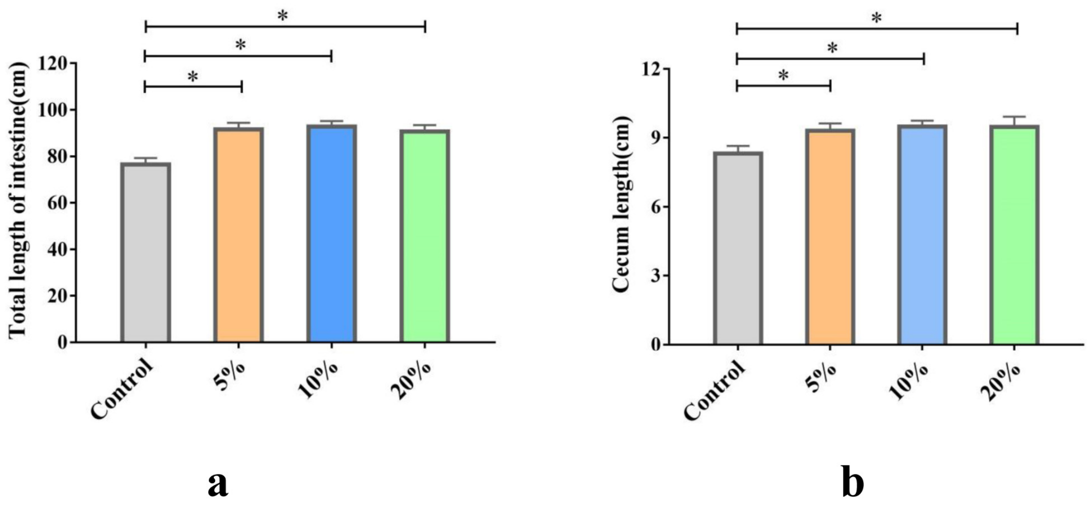

Figure S1. The effect of TWW on the length of the total intestine and cecum of chicks. (a) The average length of the total intestinal tract of the chicks in each group. (b) The average length of the cecum of the chicks in each group. Values presented as means ± SEM. * $p < 0.05$, ** $p < 0.01$, compared with the control group. NS: no significance between the indicated groups.

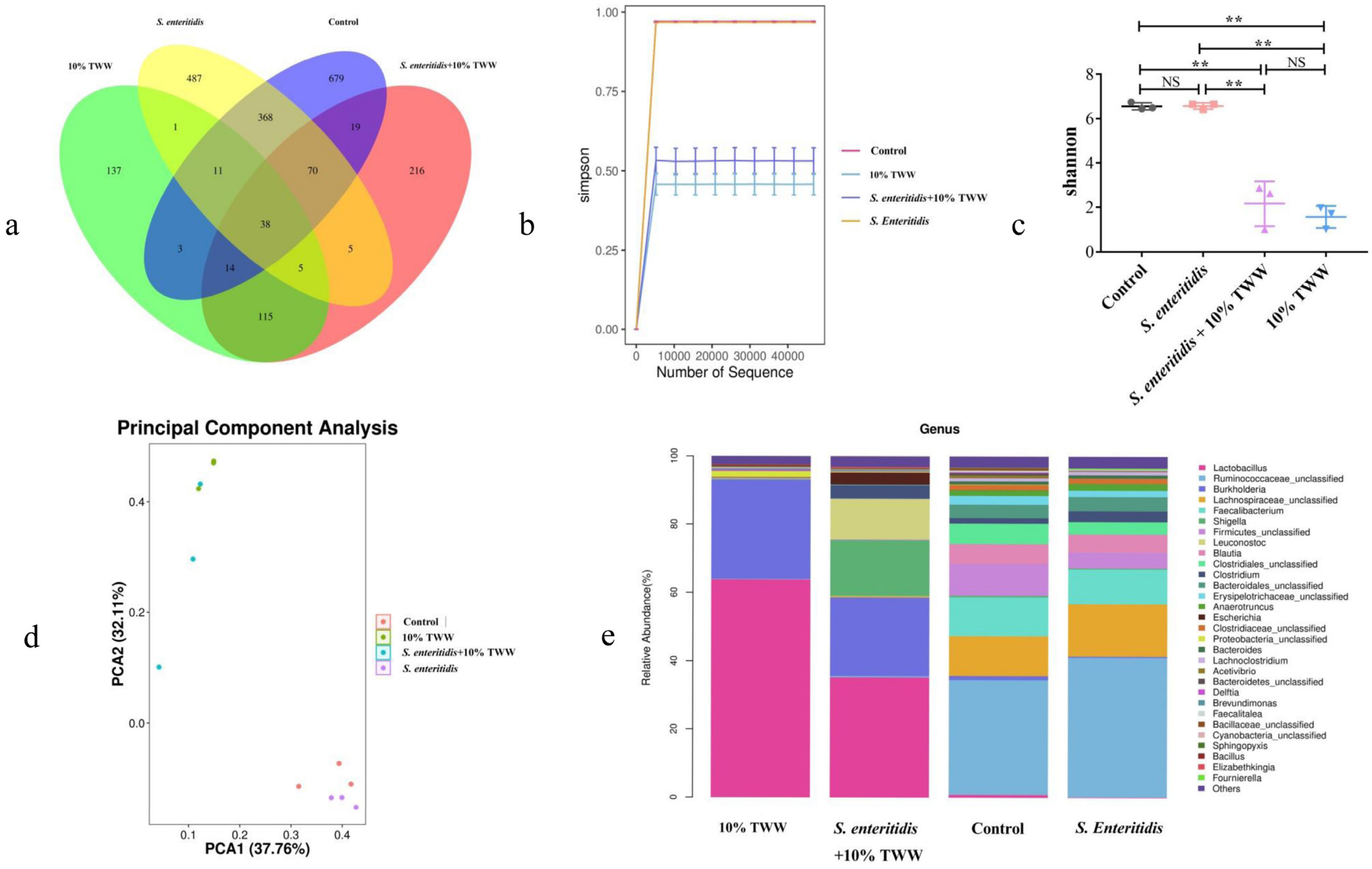

Figure S2. TWW modulates the gut microbiota diversity in chickens. (a) Venn diagram analysis of microbial flora in each group. (b,c) Comparison of alpha diversity accessed by Simpson indexes and Shannon indexes. (d) Comparison of beta diversity accessed by weighted UniFrac principal coordinates analysis (PCA). (e) Relative abundance of microbial flora at the genus level. Values presented as means ± SEM. * $p < 0.05$, ** $p < 0.01$, compared between the indicated groups. NS: no significance between the indicated groups.

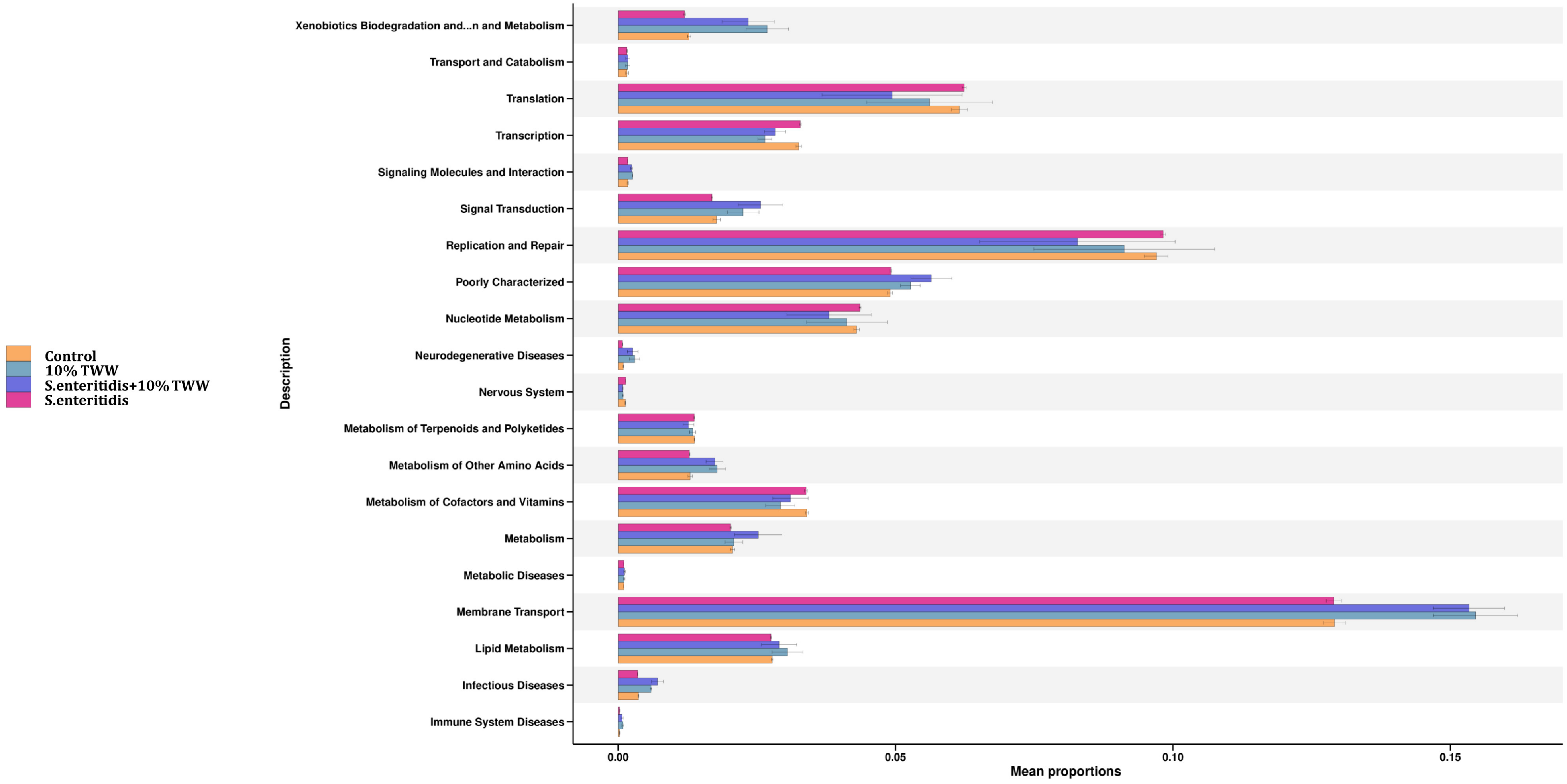

Figure S3. The functions of gut microbial community predicted by PICRUS
